# The Influence of Victim Self-Disclosure on Bystander Intervention in Cyberbullying

**DOI:** 10.3390/bs13100829

**Published:** 2023-10-09

**Authors:** Yuze Zeng, Junze Xiao, Danfeng Li, Jiaxiu Sun, Qingqi Zhang, Ai Ma, Ke Qi, Bin Zuo, Xiaoqian Liu

**Affiliations:** 1 School of Criminal Justice, China University of Political Science and Law, Beijing 102249, China; 2102040168@cupl.edu.cn (Y.Z.); 2202040210@cupl.edu.cn (J.X.); 2 School of Sociology and Psychology, Central University of Finance and Economics, Beijing 100098, China; 3 School of Sociology, China University of Political Science and Law, Beijing 102249, China; 4 The Psychological Counseling Center, China University of Political Science and Law, Beijing 102249, China; 5 Officers College of PAP, Chengdu 610213, China

**Keywords:** cyberbullying, self-disclosure, valence, victim blaming, interpersonal distance, bystander intervention

## Abstract

The frequent occurrences of cyberbullying on social platforms have sparked a great deal of social conflict, and bystander intervention plays a crucial role in preventing the escalation of cyberbullying. This research examines the impact of victim self-disclosure on bystander intervention in cyberbullying through two experimental studies. The studies collected data from March to July of 2022, utilizing a convenience sampling approach to recruit university students as experiment participants. Study 1 recruited 247 valid participants, while Study 2 recruited 522 eligible participants. The results of Study 1 indicate that the perceptible dimensions (frequency, privacy, and valence) of victim self-disclosure impact bystander intervention. Specifically, in a low privacy context, positive self-disclosure increases bystander intervention, while negative self-disclosure does the opposite. The results of Study 2 suggest that the valence of self-disclosure affects bystander intervention through the mediation of victim blaming, with interpersonal distance moderating the impact of victim self-disclosure valence on the extent of victim blaming. This moderated mediation model clarifies the psychological process by which the valence of victim self-disclosure affects bystander intervention. The findings of this study contribute to the understanding of the social psychological process behind bystander intervention, providing a scientific basis and pathway for reducing cyberbullying and fostering a harmonious online environment.

## 1. Introduction

In recent years, the phenomenon of “cyber-violence” has drawn widespread societal attention, with victims of cyberbullying often suffering substantial physical and mental harm. Cyberbullying, defined as intentionally and repeatedly attacking individuals who cannot easily protect themselves in an online environment [1], namely through the Internet, social media, or other digital tools to maliciously attack, ridicule, or harm others, has been recognized as a public health issue that severely impacts individuals’ physical and mental health globally. Previous studies have shown that nearly a third of college students have suffered from cyberbullying in the past six months [2], and 13.9% of adolescents aged 9–22 have experienced at least one incident of cyberbullying [3]. The latest systematic review study focusing on the cyberbullying prevalence rates during the COVID-19 pandemic indicates that the proportion of cyberbullying victims ranged from 8.9% to 49.2%, and this situation among Chinese adolescents has risen since the outbreak of the COVID-19 pandemic [4]. Moreover, compared to traditional bullying, cyberbullying not only leads to a series of negative psychological and behavioral issues, such as depression and anxiety, but it can also exacerbate damage to their mental health due to its occurrence in a more open online space, exposing victims to a wider audience, and even leading to problems such as self-harm and suicide [5]. How to effectively mitigate the negative influence caused by cyberbullying has become a focus of people all over the world.

Studies have shown that positive intervention from bystanders, whether in traditional contexts or online environments, can quickly and effectively prevent bullying behaviors. Support from bystanders can significantly alleviate victims’ plight [6,7]. Bystanders in the cyberbullying context refer to individuals who witness bullying incidents online but do not participate [8]. A large number of cyberbullying bystanders could either “stand up for justice” to prevent bullying incidents or potentially become “accomplices” that “fuel the flames”. Previous research found that compared to traditional bullying incidents that occur offline, the likelihood of bystander intervention in an online environment is smaller [9]. Most bystanders play the role of “indifferent observers” in incidents of cyberbullying [10,11], and some even facilitate it [9]. Among these, “victim blaming” is a common attitude among bystanders who act negatively in cyberbullying, with these bystanders believing that the reason the victim is targeted is due to certain characteristics or behaviors of the victim themselves that incite the hostility of the bully [12]. In Holfeld’s survey on the attribution characteristics of bystanders in cyberbullying incidents, it was found that most participants believed that the victims themselves caused the bullying incident, with nearly one-third attributing the cyberbullying to internal, controllable characteristics of the victim [13]. The belief in a just world theory (BJW) provides an explanation for this phenomenon, suggesting that individuals want to live in a “just world” where “good people are rewarded, bad people are punished”, believing that the world is fair can help us perceive a sense of meaning and predictability in life. However, the appearance of innocent victims shakes this belief and triggers a sense of crisis in individuals about the potential for similar injustices to befall them. In order to alleviate this sense of crisis, individuals will attribute the occurrence of unfair events to every possible “blemish” on the victim [14]. A recent eye-tracking study suggests that in cyberbullying scenarios, the victim is the character who gets more attention compared to the bully and other roles [15]. Therefore, bystanders’ impressions and views of the victims may be important factors affecting their helping behavior, and this is worthy of further exploration. It is worth noting that the bystander intervention explored in this study mainly refers to positive interventions that would help the victim.

### 1.1. The Impact of Victim Self-Disclosure (Frequency, Valence, and Privacy) on Bystander Intervention

Previous studies have found that bystanders form impressions of victims based on victim self-disclosure on social media [16,17]. Self-disclosure refers to the act of an individual revealing their thoughts, feelings, and experiences to others in interpersonal communication [18]. The content of self-disclosure includes five dimensions: honesty, purposefulness, frequency, valence, and privacy [19,20]. Among them, frequency, valence, and privacy are components that can be directly perceived by bystanders. Online bystanders can assess these by the number of individual self-disclosures, the positivity of the disclosed content, and the level of privacy (such as mentioning identification numbers and emotional states). Understanding the impact of these three perceptible dimensions of self-disclosure on bystander intervention can provide more clear and objective guidance for the online speech of victims, effectively reducing the likelihood of cyberbullying. Schacter pointed out that victim self-disclosure is a decisive factor in the cognitive, emotional, and behavioral responses of bystanders, and the appropriateness of the content presented by the victim on social media will affect the perceived responsibility of the bystanders [21]. Empirical and theoretical research has also indicated that the frequency, valence, and privacy of self-disclosure are closely related to bystander intervention: Weber et al. found that victims who disclose highly private information are less likely to receive social support and help from bystanders than those who disclose less private information [22]. Highly personalized self-disclosure can be viewed as attention-seeking behavior to some extent, leading bystanders to scrutinize and question the appropriateness of the self-disclosure content. This can possibly form a negative impression and ultimately reduce their willingness to help [17,23,24]. The social information processing theory suggests that individuals tend to notice information with strong accessibility in the network environment and react accordingly [25]. Forest and Wood pointed out that frequent sharing of personal information on social media is also considered a form of attention-seeking behavior, triggering negative judgments from bystanders [23]. Scott’s research indicates that the more negative the tweets initially posted by the victims, the more accusations they receive. When comments on negative valence tweets contain a lot of abuse, the bullying event will be evaluated by bystanders as deserving, thereby reducing their intention to intervene [26]. Based on the above, this study proposes the following hypotheses:

**Hypothesis** **1** **(H1).** *In online cyberbullying context, the frequency, valence, and privacy of victim self-disclosure will influence bystander interventions*.

### 1.2. Victim Blaming as a Mediator

Victim blaming refers to attributing the responsibility of violent victimization partially or fully to the victims themselves [27]. This is a common phenomenon in cyberbullying. Blaming the victim is both an act and an indicator of bystanders’ attitudes: that the victim deserves condemnation.

The frequency, valence, and privacy of a victim’s self-disclosure in cyberbullying can trigger different levels of blaming for the victim from bystanders. According to the impression formation theory, people’s impressions of others stem from a series of perceived “central traits,” and speech is the main way to reflect individual central traits [28]. The negativity bias theory posits that compared to positive remarks made by victims on social media, negative remarks are more likely to attract the attention of bystanders and have a greater impact on them [29]. Typically, a victim’s negative comments can leave a bad impression of hostility and pickiness on bystanders, which will magnify the influence of their belief in a just world, making them more likely to blame the victim [9]. Scott’s research on Twitter found that the more negative the tweets victims had posted, the more blame they received when they faced cyberbullying [26]. It can be speculated that the more positive victim self-disclosure in cyberbullying, the less blame from bystanders, and the more negative victim self-disclosure, the more blame from bystanders. Schacter’s research suggests that high-frequency self-disclosure, regardless of valence, increases the likelihood of an individual being blamed by others when facing cyberbullying [21]. Moreover, sharing excessively private personal information may be regarded as seeking attention or sympathy, which will incur negative evaluation from bystanders [30], leading to blaming the victim in cyberbullying.

The degree of victim blaming will further influence bystanders’ intervention behavior. According to the theory of planned behavior (TPB), individuals’ behavioral intentions and, subsequently, their actions are usually based on their attitudes towards a behavior and subjective norms. Therefore, bystanders who blame the victim will not offer help. Qualitative studies on cyberbullying also indicate that attributing blame to the victim is an important variable in predicting whether bystanders intervene or not [31,32]. The above theories and related studies point out a potential path to explain how self-disclosure affects bystander intervention: different frequencies, valences, and privacy levels of self-disclosure can trigger different degrees of victim blaming from bystanders, which in turn affects their further intervention behavior. Based on this, this study proposes the following hypothesis:

**Hypothesis** **2** **(H2).** *Victim blaming mediates the influence of victim self-disclosure (frequency, valence, and privacy) on bystander intervention*.

### 1.3. Interpersonal Distance as a Moderator

Interpersonal distance is the psychological distance between individuals in social interactions, influencing cognition, emotions, and behavior [33]. According to social identity theory, people tend to have more preference for individuals who are closer in interpersonal distance and may even be tolerant when they engage in inappropriate behavior [34,35]. Through experimental research, Fehr found that people are more likely to punish strangers who violate social norms, while they are more tolerant towards acquaintances or relatives [36]. This behavioral law of individuals is also the case in the context of the Internet. Previous studies on social media platforms like Facebook and Twitter found that people are more tolerant of the improper behavior (such as overly aggressive tweets) of those they are close to [37,38,39]. Therefore, when individuals feel closer to the victims, they may have a deeper understanding and sympathy for the victims, giving the victims who have disclosed inappropriate information more situational explanation, such as assuming the victims’ negative emotions may be caused by external environments, thereby reducing victim blaming and intervening in the cyberbullying they suffer from. Conversely, when the interpersonal distance is farther, individuals may pay more attention to general social norms and rules, becoming more likely to blame victims who frequently express negative and private information [40], thus reducing their assistance to them. Based on this, this study proposes the following hypothesis:

**Hypothesis** **3** **(H3).** *Interpersonal distance moderates the relationship between victim self-disclosure (frequency, valence, and privacy) and victim blaming*.

### 1.4. The Current Study

At present, there are limited studies on the impact of victim self-disclosure in cyberbullying on bystander intervention, and there are four main limitations: First, the samples often focus on children and middle school students, with fewer studies targeting adults; second, existing studies mostly focus on the willingness of bystanders to intervene, lacking research on their actual intervention behaviors; third, current studies mostly target a single dimension of self-disclosure, without comprehensively exploring the impact of frequency, valence, and privacy on bystander interventions. Only one study considers the impact of both valence and privacy, and it only discusses scenarios of cyberbullying where the victim is female [21]; last, no study has explored the psychological process by which specific dimensions of victim self-disclosure affect bystander interventions.

**Figure 1:**
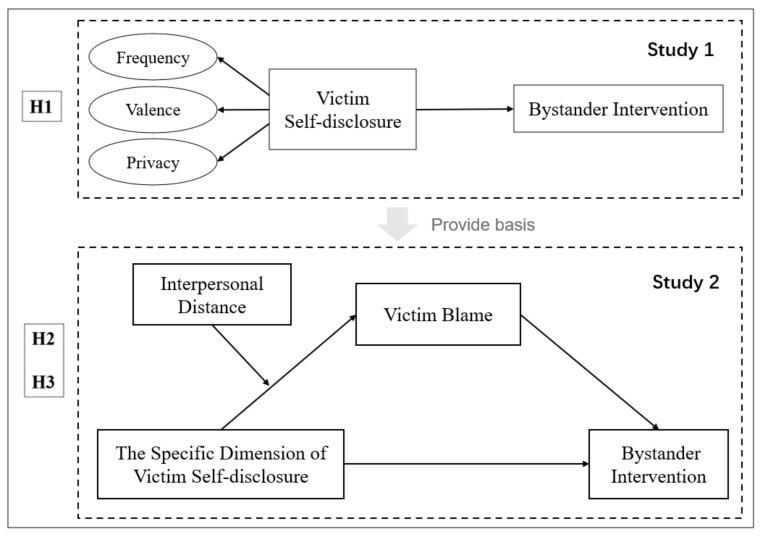
The framework of current study.

In summary, to comprehensively explore the impact of victim self-disclosure in cyberbullying on bystander interventions, this article planned to take the university student population as the research object and through two studies, clarify their relationship and its internal mechanisms: Study 1 aimed to explore the impact of the three perceptible dimensions of victim self-disclosure (frequency, valence, and privacy) on bystander interventions; Study 2, based on the results of Study 1, would further explore the psychological process of the impactful specific dimensions of victim self-disclosure on bystander interventions. The aim is to provide a foundation for reducing cyberbullying incidents and fostering a more harmonious and civilized online environment. The framework of these studies is shown in Figure 1.

## 2. Study 1: The Impact of the Frequency, Valence, and Privacy of Victim Self-Disclosure in Cyberbullying on Bystander Interventions

### 2.1. Purpose

The purpose of the study was to explore the impact of the frequency, privacy, and valence of victim self-disclosure in cyberbullying on bystander intervention.

### 2.2. Method

#### 2.2.1. Participants

This study used convenience sampling among university students because they are known for their high frequency of Weibo usage (Weibo is regarded as ‘Chinese Twitter’, a popular social networking platform where users can share messages, images, and multimedia with followers or strangers. It supports interactions like comments, upvotes, and reposts, which can promote social engagement, information sharing, and online discussion among users), making them more familiar with cyberbullying online. The study was conducted at China University of Political Science and Law, Beijing, from March to April of 2022.

First, we recruited undergraduate and master’s students with Weibo use experience through an online advertisement; a total of 296 university students in Beijing were enrolled in this study.

Second, we excluded those with physical illness or mental health issues or with a background in psychology (*n* = 16). Because individuals with physical illness or mental health issues might exhibit abnormal behavior due to their unique circumstances, participants with a background in psychology may have a heightened awareness of the study’s objectives and measures. Then, we utilized stratified random grouping to divide the subjects into different experimental treatments based on their age, Weibo usage frequency, agreeableness, etc.

Third, before formal data analysis, we excluded participants with quick or regular responses through pre-analysis (*n* = 33) since too short response times may suggest that participants did not adequately consider the questions or provided random answers, potentially affecting the results.

We collected informed consent from all participants before they started the study. At last, a total of 247 participants were included in the final data analysis. Among them, there were 101 males and 146 females, with ages ranging from 18 to 29 years old (*M* = 23.18, *SD* = 2.9).

#### 2.2.2. Design

This study adopted a three-factor 2 (Self-disclosure frequency: High, Low) × 2 (Self-disclosure privacy: High, Low) × 2 (Self-disclosure valence: Positive, Negative) between-subjects design, with participants divided into eight groups. The frequency, valence, and privacy of self-disclosure of victims in cyberbullying were all independent variables, while the intensity of the participants’ (bystanders) intervention behavior was the dependent variable. There were 31 people in the high frequency-high privacy-positive disclosure group and the low frequency-high privacy-negative disclosure group, 35 people in the high frequency-low privacy-positive disclosure group, and 30 people in each of the remaining five groups.

#### 2.2.3. Procedure

Drawing on the study by Schacter et al. (2016), we initially created experimental materials containing eight scenarios [21]. Participants were then randomly assigned to different scenarios. Upon arriving at the laboratory, participants were asked to fill out basic information, such as gender, age, frequency of Weibo usage, and the agreeableness subscale of the Big Five Personality Questionnaire, in order to control for potential extra variables. Prior to the experiment, we informed participants that this was a survey about Weibo usage habits and asked them to carefully read the instructions, review the experimental materials, and report their personal feelings about the material for the researchers to evaluate the validity of the material. Finally, participants were invited to complete the Bystander Intervention Questionnaire.

#### 2.2.4. Materials

##### Cyberbullying Scenarios

The study used simulated scenarios and asked participants to imagine that they inadvertently witnessed a cyberbullying incident while browsing Weibo. Participants were then requested to carefully review Victim’s Weibo posts and answer questions about their genuine feelings and behavioral tendencies [41]. The setup of the cyberbullying scenarios was adapted from the materials used in Schacter et al.’s (2016) research and was further modified according to the Chinese context and real-life situations [21]. The scenario materials were divided into eight types according to the frequency (high, low), privacy (high, low), and valence (positive, negative) of victim self-disclosure. The researchers ensured consistency in the length and structure of materials across different scenarios. The contextual background of the scenarios remained consistent across all eight groups, including background and profile pictures of victims’ Weibo, links to music, thumbnails of videos, and so on. Under high-frequency conditions, Victim A posted three Weibo posts in a day, while under low-frequency conditions, Victim A posted three Weibo posts within a month. In terms of low privacy, Victim A expressed his/her opinion on a TV program in the most recent Weibo post, while in high privacy, Victim A shared feelings about his/her romantic relationship in the most recent Weibo post. In positive valence conditions, the most recent Weibo post of Victim A started with “I like...”, while in negative valence conditions, the most recent Weibo post started with “I hate...”.

**Figure 2:**
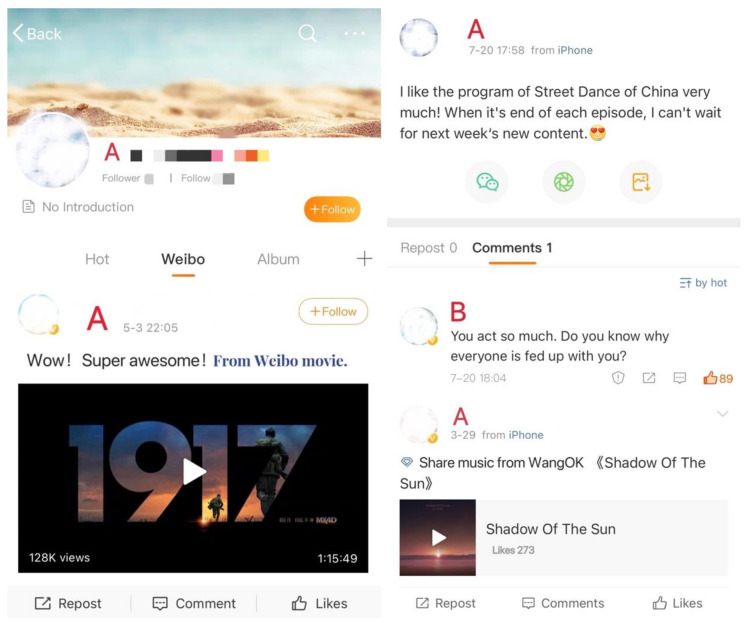
Material for Cyberbullying Scenario with Low Frequency, Low Privacy, and Positive Self-Disclosure. Note. ‘A’ represents the victim, while ‘B’ signifies the bully.

Here is an example: the specific content of the material for the cyberbullying scenario where the victim exhibits low frequency, low privacy, and positive self-disclosure is shown in Figure 2.

##### Bystander Intervention

We adapted the questionnaire on bystander behavior developed by Bastiaensens et al. [42] to measure the likelihood of participants actively helping the victim of cyberbullying. In previous studies, this scale is often used directly or adapted to measure bystanders’ tendency to help victims and has an acceptable reliability consistency coefficient above 0.73 [42,43]. The questionnaire includes six items, which are “leaving a comment in support of victim A”, “privately comforting and supporting victim A”, “asking bully B to stop”, “bully B with similar comments”, “privately chat with B and points out B’s mistakes” and “reporting bully B’s comments on Weibo”. The questionnaire uses a Likert scale scoring from 1 “totally unlikely” to 5 “very likely”. The higher the score, the higher the level of intervention from the participant as a bystander. The Cronbach α coefficient for the bystander intervention questionnaire in this study was 0.79.

##### Frequency of Weibo Usage

The frequency of Weibo usage was measured by a question: “Please select the description that most closely matches your Weibo usage frequency.” This question uses a Likert scale scoring from 1 “never” to 5 “always”. The higher the score, the higher the frequency of Weibo usage.

##### Agreeableness

We used the Agreeableness subscale from Caprara’s revised Big Five Personality Questionnaire to measure the participants’ level of agreeableness [44,45] with adequate validity in Chinese [46] and controlled it as an extraneous variable. The Agreeableness subscale includes 12 items; each scored on a five-point scale from “strongly disagree” to “strongly agree”. The higher the total score, the higher the degree of agreeableness in participants. The Cronbach *α* coefficient for this scale in this study was 0.81.

##### Cyberbullying Scenarios Validity Assessment

Referring to the study by Huang et al. [47], we designed a questionnaire to measure the subjective feelings of the participants towards the experimental materials, including aspects of frequency, privacy, and valence. The questionnaire uses a Likert five-point scale and contains three items which are “How do you rate the frequency of A’s Weibo posts?”, “How do you rate the privacy of A’s Weibo posts?”, “How do you rate the positivity of A’s Weibo posts?”, with scores ranging from 1 “very low” to 5 “very high”. The measurement of these issues is mainly used to test whether the manipulation of the independent variables is successful.

### 2.3. Results

#### 2.3.1. Analysis of the Control Variables and Cyberbullying Scenarios

Prior to the formal analysis, Shapiro-Wilk tests revealed that all variables had a normal distribution. Firstly, this study conducted a difference test on the gender, age, Weibo usage frequency, and agreeableness of the participants in different groups to clarify whether the above control variables would have an impact on the dependent variables. A chi-square test was conducted on different groups and genders. The results showed that there was no significant difference in gender distribution between the high-frequency group (49 males, 77 females) and the low-frequency group (52 males, 69 females) of victim self-disclosure, *χ*^2^ = 0.426, *df* = 1, *p* = 0.514; the high-privacy group (52 males, 70 females) and the low-privacy group (49 males, 76 females) showed no significant difference in gender distribution, *χ*^2^ = 0.299, *df* = 1, *p* = 0.584; the difference in gender distribution between the group with positive self-disclosure (54 males, 72 females) and the group with negative self-disclosure (47 males, 74 females) was not significant, *χ*^2^ = 0.411, *df* = 1, *p* = 0.521. An analysis of variance was conducted with the frequency, valence, and privacy of victim self-disclosure as independent variables and age, Weibo usage frequency, and agreeableness as dependent variables. The results showed that the main effects of frequency, privacy, and valence on age, Weibo usage frequency, and agreeableness were not significant, and there were no significant interaction effects. The specific data results are shown in Table 1. These results indicate that there is no significant difference in the scores of the control variables among different groups, and further analysis can be conducted.

The study conducted a manipulation check for the validity of the experimental scenarios, the results of which show that the manipulation of independent variables in the experimental materials is effective. Specifically, participants in the high-frequency group perceived the frequency of victim self-disclosure (*M* = 3.94, *SD* = 0.69) to be significantly higher than the perceived frequency score of participants in the low-frequency group (*M* = 2.25, *SD* = 0.75), *t* (245) = 18.65, *p* < 0.001, *Cohen’s d* = 2.35. Participants in the high-privacy group rated the privacy of victim self-disclosure (*M* = 3.55, *SD* = 0.89) significantly higher than the privacy score of participants in the low-privacy group (*M* = 2.30, *SD* = 0.91), *t* (245) = 10.87, *p* < 0.001, *Cohen’s d* = 1.39. Participants in the positive self-disclosure group rated the positivity of victim self-disclosure (*M* = 3.52, *SD* = 0.79) significantly higher than the positivity score of participants in the negative self-disclosure group (*M* = 2.43, *SD* = 0.75), *t* (245) = 11.17, *p* < 0.001, *Cohen’s d* = 1.42.

#### 2.3.2. Bystander Intervention

**Figure 3:**
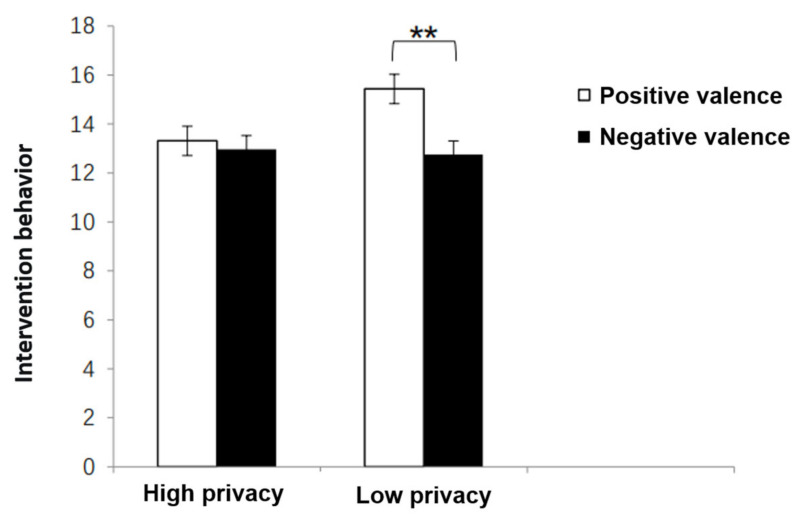
Participant’s Intervention Behavior under Different Experimental Scenarios. Note. Error bars represent standard error, ** *p* < 0.01.

A variance analysis was conducted with the degree of the participant’s intervention behavior as the dependent variable and the frequency, privacy, and valence of victim self-disclosure in the cyberbullying scenario as the independent variables. The results showed the main effect of frequency was not significant, *F* (1, 239) = 2.943, *p* = 0.09; the main effect of privacy was not significant, *F* (1, 239) = 2.611, *p* = 0.11; the main effect of valence was significant, *F* (1, 239) = 6.58, *p* = 0.01, *ƞ*^2^*_p_* = 0.03. The intensity of bystander intervention when victim self-disclosure is positive (*M* = 14.44, *SD* = 4.76) is significantly higher than the intervention intensity for victims of negative self-disclosure (*M* = 12.86, *SD* = 4.57). In addition, the interaction between privacy and valence was significant, *F* (1, 239) = 3.98, *p* = 0.04, *ƞ*^2^*_p_* = 0.02, as seen in Figure 3. Simple effect analysis found that under the condition of high privacy self-disclosure of the victim, the valence of self-disclosure did not affect the intensity of the participant’s intervention. However, under the condition of low privacy self-disclosure of the victim, the intensity of the participant’s intervention for victims of positive self-disclosure (*M* = 15.43, *SD* = 0.58) was significantly higher than the intervention intensity for victims of negative self-disclosure (*M* = 12.75, *SD* = 0.60).

### 2.4. Discussion

The results of Study 1 indicated that among the three dimensions of victim self-disclosure, valence had a significant effect on bystander intervention, and there was a significant interaction between valence and privacy. Specifically, when the privacy of victim self-disclosure was low, positive self-disclosure could increase bystander intervention compared to negative self-disclosure, which partially validated Hypothesis H1. Therefore, in the Chinese cultural context, in the event of cyberbullying, low privacy is the situational basis for bystander intervention, while the valence of victim self-disclosure is the key factor influencing whether bystanders intervene. So, why do positive and negative self-disclosures by the victim have different effects on bystander intervention in the same low privacy situation? What is the specific psychological process of this influence? The following Study 2 will examine this.

## 3. Study 2: The Impact of Victim Self-Disclosure Valence on Bystander Intervention and Its Psychological Process

### 3.1. Purpose

Based on Study 1, this study explores the psychological process underlying the impact of self-disclosure valence on bystander intervention under conditions of low victim self-disclosure privacy. This study tested the moderated mediation model proposed in the review section.

### 3.2. Method

#### 3.2.1. Participants

The participant’s recruitment procedure and screening conditions for Study 2 were the same as those of Study 1. This study was conducted at China University of Political Science and Law from June to July of 2022, using convenience sampling. All participants gave informed consent before the experiment. A total of 522 valid participants were recruited, including 232 males and 290 females, aged between 17 and 31 years old (*M* = 21.06, *SD* = 2.61).

#### 3.2.2. Design

This study adopted a 2 (Valence: Positive, Negative) × 2 (Interpersonal Distance: Far, Close) between-subjects design, with the valence of victim self-disclosure and the interpersonal distance between the victim and the bystander as independent variables. The dependent variable was the intensity of the bystander intervention behavior, with the degree of the bystander’s blame on the victim as the mediating variable. Finally, there were 132 participants in the positive valence-far interpersonal distance group, 132 in the positive valence-close interpersonal distance group, 133 in the negative valence-far interpersonal distance group, and 125 in the negative valence-close interpersonal distance group.

#### 3.2.3. Procedure

Based on the cyberbullying scenario developed in Study 1, participants were randomly assigned to one of the four different scenarios. Prior to the experiment, participants were asked to complete the same control variable scales as in Study 1. In order to further minimize the interference of irrelevant factors, Study 2 added self-efficacy and experiences of cyberbullying as control variables, as prior research found that an individual’s self-efficacy and past experiences of cyberbullying significantly affect their altruistic behavior in online scenarios [48,49]. Before the experiment, participants were informed that “this is a survey about the experience of using Weibo” and asked to assess their feelings about the materials and their interpersonal distance from the victim after carefully reading the instructions and browsing the experimental materials. Subsequently, participants filled out a questionnaire on victim blame, and finally, they were asked to “post their own comments” on the victim’s Weibo. At the end of the experiment, the experimenter evaluated the comments they left.

#### 3.2.4. Materials

##### Cyberbullying Scenarios

Study 2 also used the simulated scenario form in Study 1. The manipulation of low privacy and different valence self-disclosure of the victim was the same as in Study 1.

##### Bystander Intervention

Study 1 used a bystander intervention scale to measure the likelihood of the participant helping the victim. In order to further enhance the ecological validity of the dependent variable measurement, Study 2, referring to the design of the previous study [50], asked participants to “post their own comments” on the victim’s Weibo after browsing the cyberbullying scenario materials. This measurement method can more directly and objectively reflect the intervention behavior of bystanders. For example, Victim A posted on Weibo, “I hate ‘Street Dance’ so much, I have to wait a week for updates after each episode”, and Bully B left a comment under the post saying, “No wonder everyone hates you, it’s an insult to the show for people like you to watch”, this is a typical cyberbullying situation. The study asked participants to leave their own comments after watching, and the results of each participant’s comment were rated by two trained investigators who were unaware of the purpose of the study and the conditions of the participants. Specifically, the investigators used a 5-point scoring system to assess the participants’ comments: 1 indicated “negative”, including explicit negative comments on victim A, such as “You should study hard instead of watching these things all the time”; 2 indicated “a bit negative”, referring to responding to this event in a negative way, but not explicitly targeting victim A, for example, “Aren’t all variety shows like this”; 3 indicated “neutral”, pointing to other aspects unrelated to victim A, such as “Rainy day, suitable for eating hot pot”; 4 indicated “a bit positive”, positive comments about victim A, but can’t completely help victim A avoid bullying, such as “Thanks for sharing, don’t mind those negative comments”; 5 indicated “positive”, which could clearly support victim A or help her deal with bullying, such as replying to bully B “She is expressing her own feelings here, she can say whatever she wants, you can’t bully her like this”. The higher the rating, the stronger the intervention behavior of the participant. The interrater reliability of this material was 0.83.

##### Victim Blaming

Referring to the 5-item measure used by Schacter et al. (2016) in their study (e.g., “A got what they deserved”) [21], the degree to which participants attributed the cyberbullying event to the victim themselves was measured. Participants were asked to rate on a five-point scale from “strongly disagree” to “strongly agree”. In this study, the Cronbach’s *α* coefficient for this scale was 0.80.

##### Interpersonal Distance

The interpersonal distance was manipulated through the introduction of the personal homepage of Weibo, and the relationship between the test participant and the victim was explained in the instructions. The test participants in the close interpersonal distance group were told that the victim was a good friend, and they would see that their relationship with the victim was “mutually followed”. The test participants in the far interpersonal distance group were told that the victim was a stranger, and they would see that they did not follow each other with the victim. The rest of the personal information (such as gender, avatar, region, etc.) was blurred. At the end of the experiment, the test participants were asked to evaluate “the current interpersonal distance between you and the Weibo poster” using a 5-point Likert scale. Scores from 1 to 5 represented the interpersonal distance being “very close”, “relatively close”, “neutral”, “relatively far”, and “very far”, respectively. This measurement was mainly used to check whether the manipulation of the interpersonal distance variable was successful, and the results showed that the manipulation of this variable was effective.

##### Weibo Usage Frequency and Agreeableness

The same questionnaires as in Study 1 were used for the measurement.

##### Self-Efficacy

The General Self-Efficacy Scale, compiled by Schwarzer et al. and revised by Wang et al. [51], was used. It included 10 items, scored on a 5-point scale from “completely disagree” to “completely agree”. The higher the score, the stronger the individual’s sense of self-efficacy. The Cronbach’s *α* coefficient for this scale in Study 2 was 0.91.

##### Cyberbullying Experiences

The Cyberbullying Scale, compiled by Topcu and Erdur-Baker and revised by Chu and Fan [52], was used to assess the frequency with which the participants had been victims of cyberbullying in the past six months. The scale included 14 items, with a 4-point scoring scale representing “never encountered”, “once”, “2–3 times”, and “more than 3 times”. The Cronbach’s *α* coefficient for this scale in Study 2 was 0.88.

### 3.3. Results

#### 3.3.1. Descriptive Statistics and Correlation Analysis

The descriptive statistics and Pearson correlation coefficients of the variables in this study are presented in the table below. As shown in Table 2, victim blaming is significantly negatively correlated with bystander intervention (*r* = −0.37, *p* < 0.01). At the same time, the frequency of Weibo usage is significantly positively correlated with bystander intervention (*r* = 0.14, *p* < 0.01) and significantly negatively correlated with victim blaming (*r* = −0.28, *p* < 0.01). Self-efficacy is significantly positively correlated with victim blaming (*r* = 0.10, *p* < 0.05) and negatively correlated with cyberbullying experience (*r* = −0.10, *p* < 0.05). The pairwise correlations between other variables are not significant, so only Weibo usage frequency and self-efficacy are controlled in subsequent analyses. Table 2 presents the descriptive statistics and correlation matrix for each variable (*n* = 522).

#### 3.3.2. Mediation Effect Analysis

This study used the macro file PROCESS in SPSS, selected model 4, with the valence of victim self-disclosure as the independent variable X (assigned 1 = positive disclosure, 2 = negative disclosure), bystander intervention as the dependent variable Y, and victim blaming as the mediator variable M. Weibo usage frequency and self-efficacy were taken as control variables. The mediation role of the model was verified through a bootstrap sampling procedure, with the number of bootstrap samples set at 5000, using a bias-corrected 95% confidence interval.

**Figure 4:**
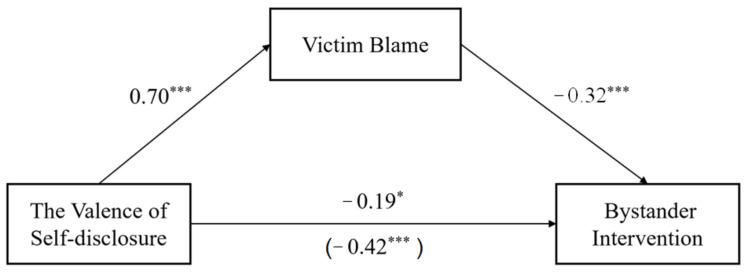
Path diagram of the relationship between victim self-disclosure valence and bystander intervention. Note. * *p* < 0.05; *** *p* < 0.001.

The results showed that the valence of victim self-disclosure effectively predicted bystander intervention (*β* = −0.19, *p* = 0.03) and victim blaming (*β* = 0.70, *p* < 0.001), and victim blaming negatively predicted bystander intervention (*β* = −0.32, *p* < 0.001). The regression coefficients of each path are shown in Figure 4.

The results of the mediation effect analysis are shown in Table 3, with an indirect effect of −0.19 (95%CI = [−0.26, −0.13]) when victim blaming is used as a mediator. This suggests that victim blaming mediates the effect of the valence of victim self-disclosure on bystander intervention.

#### 3.3.3. Moderation Effect Analysis

**Figure 5:**
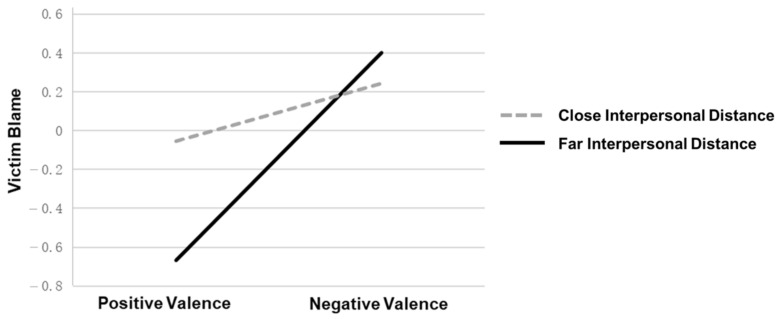
Moderation Effect of Interpersonal Distance on the Impact of Victim Self-Disclosure Valence on Victim Blaming.

Using the PROCESS plug-in for SPSS 26.0 and following the Bootstrap method proposed by Hayes (2013) for testing the moderating effect [53], Model 7 was selected. With the valence of victim self-disclosure as the independent variable X (assigned 1 = positive disclosure, 2 = negative disclosure), the bystander’s intervention behavior as the dependent variable Y, victim blaming as the mediating variable M, interpersonal distance as the moderating variable W (assigned 1 = far interpersonal distance, 2 = close interpersonal distance), and Weibo usage frequency and self-efficacy as control variables. The regression analysis results showed that the valence of victim self-disclosure (*β* = 0.69, *p* < 0.001) and interpersonal distance (*β* = 0.23, *p* = 0.002) can effectively predict the degree of victim blaming by the participants. The interaction term of the two has a significant regression coefficient on victim blaming (*β* = −0.77, *p* < 0.001), indicating that interpersonal distance can moderate the effect of the valence of victim self-disclosure on participant’s victim blaming. Further simple effect analysis showed that for the far interpersonal distance group, when the valence of the victim self-disclosure changed from positive to negative, the degree of increase in the victim blaming score of the participants was larger (*effect* = 1.07, *SE* = 0.10, 95%CI [0.87, 1.27]); for the close interpersonal distance group, when the valence of the victim self-disclosure changed from positive to negative, the degree of increase in the victim blaming score of the participants was smaller (*effect* = 0.30, *SE* = 0.10, 95%CI [0.09, 0.50]). As shown in Figure 5, the moderating effect is significant.

### 3.4. Discussion

Study 2 found that the valence of victim self-disclosure indirectly influenced the bystander intervention through the degree of blaming the victim. This suggested that the more negative the victim’s self-disclosure was, the more blame they received, making it less likely for bystanders to intervene in the cyberbullying they experienced. These results validated Hypothesis H2 and confirmed the applicability of theories such as the belief in a just world theory and impression formation theory in cyberbullying situations. This finding was consistent with the results of research conducted by Scott in 2019 [26]. At the same time, Study 2 also found that interpersonal distance moderates the effect of self-disclosure valence on blaming the victim, confirming Hypothesis H3. Specifically, compared to victims who have a farther interpersonal distance from bystanders, those with a closer interpersonal distance receive less blame for their negative self-disclosures and receive more help from bystanders. These results validated the social identity theory, which suggests that people have more positive impressions of individuals who are psychologically closer to themselves and are more willing to provide help when they encounter difficulties. These results were also consistent with previous qualitative research conclusions on bystander behavior in cyberbullying [31,32].

## 4. General Discussion

Cyberbullying, as an extension of traditional bullying in cyberspace, has attracted much attention due to its wide-ranging harm, strong propagation, significant social impact, and difficulty in control. The vast number of bystanders in the online world play a vital role in curbing cyberbullying and alleviating the pain of victims. Based on the largest Internet social media platform in China (Weibo), this study constructed a “cyberbullying scenario paradigm” and comprehensively explored the influence and underlying process of the three perceptible dimensions in victim self-disclosure on bystander intervention. The study clarified the specific conditions under which victim self-disclosure influences the bystander intervention and revealed the potential psychological process behind the “victim-blaming” phenomenon in cyberbullying incidents, based on classic social psychological theories such as the belief in a just world theory, impression formation theory, and social identity theory. The results of the study indicate that in cyberbullying incidents, when victims engage in low privacy self-disclosure, positive self-disclosure can significantly improve bystander intervention compared to negative self-disclosure. Victim blaming plays a mediating role in the impact of victim self-disclosure valence on bystander intervention, while interpersonal distance moderates the effect of victim self-disclosure valence on victim blaming.

### 4.1. The Impact of Victim’s Self-Disclosure Frequency, Valence, and Privacy on Bystander Intervention

Study 1, by manipulating the frequency, privacy, and valence of victim self-disclosure, we examined their impacts on bystander intervention. The results found a significant main effect of valence and a significant interaction between valence and privacy. In low-privacy situations, compared to those with negative self-disclosures, individuals with positive self-disclosures are more likely to gain support and help from bystanders. This result is consistent with the findings of Weber et al. [22] and Schacter et al. [21]. This could be because, according to impression management theory, people have a tendency to manage their impressions positively, displaying a good self-image to others [54]. This creates positive behavioral expectations when others present their self-image. Negative comments posted by the victims on social platforms undoubtedly violate these expectations, which will attract the close attention of bystanders. Coupled with the Chinese social value orientation that advocates “positive energy” and rejects negative comments in public spaces [55], negative self-disclosure may cause strong disgust among bystanders. Therefore, compared to victims with positive self-disclosure, individuals with negative self-disclosure are more likely to leave a positive impression on bystanders and, hence, are less likely to receive their help. On the other hand, traditional Chinese cultural values emphasize a modest and benevolent style of behavior [56]. Therefore, highly private self-disclosure is likely to be labeled as “showy”, reducing the likelihood of bystanders providing help, and only when the self-disclosure is of low privacy and positive do bystanders tend to intervene. It is worth noting that the main effects of frequency and privacy in this study are not significant, which is not consistent with previous research findings [22,23]. This may be because, at the time of previous research, the use of social media was not as widespread as it is now, and under the impact of the current era of social media, individuals are used to high-privacy and high-frequency self-disclosure from others. As long as the content they post does not affect the reading experience of bystanders (such as posting negative information on social media will trigger negative emotional experiences in readers [57]), it would not trigger changes in bystander attitudes or behaviors.

### 4.2. Psychological Process of Victim Self-Disclosure Valence on Bystander Intervention

Study 2, Based on the results of Study 1, explored the psychological process by which the valence of self-disclosure under low privacy conditions influences bystander intervention. It was found that negative self-disclosure led to bystander victim blaming, which in turn reduced bystander intervention. The findings align with Scott’s quantitative research and conclusions from qualitative studies on cyberbullying [26,31,32]. These results potentially illuminate the specific psychological process by which a victim’s self-disclosure influences bystander intervention, suggesting that victims posting negative comments online might trigger blame from bystanders, subsequently diminishing their likelihood of positive intervening. The results also offer a psychological perspective explanation of the prevalent and misguided “victim-blaming” phenomenon observed on current social media platforms.

On the one hand, according to belief in a just world theory [14], individuals tend to believe the world is just and that misfortunes occur due to one’s inappropriate actions. Such a belief provides individuals with a heightened sense of security and enhances their feeling of control over their lives [58]. However, the presence of an innocent victim challenges this notion, making individuals question whether they could face a similar situation. Therefore, people tend to attribute cyberbullying events to victims and try to condemn and amplify possible defects in victims to alleviate their sense of loss of control and anxiety when facing unjust circumstances. The negative information previously posted by the victim on Weibo provides a “target” for bystanders to stigmatize and blame them. On the other hand, bystander intervention is, in fact, a moral act of helping others. According to theories related to moral disengagement, blaming attribution, as one of the important moral disengagement mechanisms, can reduce people’s perceived responsibility and victims’ pain in negative events, relieve the moral pressure on bystanders [59], and thus ignore cyberbullying events. The same pattern has been found in case studies on traditional campus bullying, where both perpetrators and bystanders stigmatize victims based on their overt (such as height, appearance, family situation, etc.) or implicit characteristics (such as behavior, sexual orientation, etc.), and blame them to justify their bullying or inaction behavior morally [60].

Study 2 also found that interpersonal distance moderated the impact of victim self-disclosure valence on the degree of victim blaming, thereby influencing their intervention behavior. This result is consistent with many previous studies on how interpersonal distance affects individual attitudes and behaviors, and people are more willing to accept and help those who are closer to them in interpersonal distance [61,62]. The result may be due to the dual factors of society and culture. Firstly, theories such as social identity theory and proximity effect suggest that people identify more with individuals who are closer to them in interpersonal distance [34,63]; even for temporarily formed groups generated by random sampling, individuals still prefer people who are closer in interpersonal distance [64]. Therefore, when faced with victims who have posted negative information, bystanders’ preference for victims who are closer in interpersonal distance mitigates the adverse impact of negative information on impressions and reduces the likelihood of blaming them. Secondly, compared with the cultural background of Western societies, in China’s cultural values, people pay more attention to maintaining good interpersonal relationships, even going as far as to take actions such as relaxing rules or accommodating others for the sake of harmonious relationships [65]. A very typical phenomenon is the promotion of the “Principle of Kin Concealment” behavior in ancient Chinese judicial practice [66]. Therefore, in the cultural context of China, interpersonal distance is an important moderator of the impact of self-disclosure valence on victim blaming. Close interpersonal distance buffers the negative impact of negative self-disclosure. Study 2 confirmed hypotheses 2 and 3 of this study by verifying the moderated mediation model, clarifying the internal process by which the valence of victim self-disclosure affects bystander intervention.

### 4.3. The Application Value of the Study

This study not only theoretically reveals how victim self-disclosure can affect bystander intervention. More importantly, the findings of this study provide valuable insights for increasing altruistic behavior online and reducing the negative impact of cyberbullying on individuals in practice. First, network safety literacy education is necessary and can provide individuals with self-protection skills and awareness of the Internet. The education should include understanding the nuances of online users’ self-disclosure and its potential negative influences. In an era where digital interactions are ubiquitous, disseminating the above knowledge to the public is paramount [67]. Second, social media software should also remind users to be cautious about protecting their privacy when sharing life experiences and personal views. For users, utilizing privacy settings to limit who can view or comment on their content can serve as an effective safety precaution; it’s not advisable to excessively expose private information and negative emotions on these platforms [68]. Third, we need to educate the public on the absurdity of “victim-blaming” and emphasize that the fault of cyberbullying lies not with the victims but with the perpetrators. Public media should criticize the act of victim blaming and provide relevant educational and scientific popularization resources to help people understand the situation and feelings of victims, thereby arousing bystanders’ understanding, sympathy, and assistance intention for the victims. Fourth, the results of this study affirm the importance of interpersonal relationships in individuals’ social life. When cyberbullying occurs, our friends and family are most likely to provide us with enough social support. We should learn to seek help from important others actively. Furthermore, we encourage users to establish more genuine interactions with other people on social media platforms. For instance, when reposting someone else’s social media post, instead of simply commenting, “Looks good,” consider leaving a more friendly and genuine comment like, “This photo truly captures the wonderful light in that moment. Thank you for sharing this with us.” Kunz and Seshadri’s research found that genuine and in-depth online interactions can enhance trust and a sense of community among users, thereby increasing intimacy [69] and reducing the risk of being subjected to online violence.

### 4.4. Limitations and Future Directions

In summary, this study still has the following limitations: Firstly, the data collected through questionnaire surveys may be subject to social desirability bias, where individuals might be more inclined to show positive intervention intentions and behaviors. Future studies could consider designing experiments with higher ecological validity to measure bystander intervention. For example, following the experimental design of Barlińsk et al. [70], a virtual chat room could be created in which a “friend” sends the participant offensive images and information about a classmate to observe whether the participant would intervene. Secondly, this study only investigated the effects of perceptible dimensions in victim self-disclosure (frequency, privacy, and valence) and did not study other dimensions (such as honesty and intentionality). Although honesty and intentionality are internal thoughts when the victim discloses themselves and the bystander’s perception may not be direct or may deviate from these, these two dimensions may also affect the subsequent behavior of bystanders. Future research could construct more comprehensive cyberbullying scenarios and conduct studies on this. Thirdly, we used a translated version of the bystander intervention scale from English, and there is no prior research justifying the reliability and validity of this Chinese version. In the future, we need to conduct a more in-depth study on the reliability and validity of this questionnaire in Chinese subjects. Fourthly, when exploring the impact of interpersonal distance, this study only distinguished between “mutually followed” friends and strangers on social media platforms. Future research could make more detailed divisions (such as adding categories like “relatives”, “disliked people”, etc.). Lastly, this study only focused on the university student population, and the conclusions drawn may not apply to other age groups or demographics. For instance, previous research has shown significant behavioral differences in cyberbullying across groups with varying levels of education and age ranges [71]. Future research might consider using extensive and diverse samples to provide a more comprehensive perspective on bystander behavior of cyberbullying.

## 5. Conclusions

This study found through two experiments that in cyberbullying when victim self-disclosure content has low privacy, bystanders are more likely to help victims who engage in positive self-disclosure than those who engage in negative self-disclosure. The psychological process behind this phenomenon is that victims who engage in negative self-disclosure elicit blame from bystanders, thereby reducing their intervention behavior. However, a closer interpersonal distance can alleviate the blame from bystanders caused by negative self-disclosure, thereby increasing the possibility of bystander intervention.

## Figures and Tables

**Table 1:** Analysis of Variance of Control Variables for Participants in Different Groups (*N* = 247).

Dependent Variables	Independent Variables	*M*	*SD*	*F*	*p*
Age	Frequency	23.26	3.11	0.20	0.65
		23.10	2.68		
	Privacy	23.34	2.79	0.64	0.42
		23.03	3.01		
	Valence	23.44	2.74	2.08	0.15
		22.92	3.04		
Weibo Usage Frequency	Frequency	3.25	1.26	0.59	0.45
		3.38	1.40		
	Privacy	3.25	1.33	0.56	0.46
		3.37	1.34		
	Valence	3.28	1.41	0.14	0.71
		3.35	1.25		
Agreeableness	Frequency	36.42	15.17	0.34	0.56
		35.51	13.10		
	Privacy	36.83	14.58	0.79	0.37
		35.14	13.77		
	Valence	35.66	14.423	0.09	0.76
		36.31	13.94		

**Table 2:** Descriptive statistics and correlation coefficient matrix of each variable (*N* = 522).

Variables	*M*	*SD*	1	2	3	4	5	6
1. Bystander intervention	3.13	0.83	—					
2. Victim Blaming	10.92	3.68	−0.37 **	—				
3. Cyberbullying Experience	18.57	6.15	0.01	0.05	—			
4. Frequency of Weibo Usage	3.94	1.34	0.14 **	−0.28 **	−0.01	—		
5. Agreeableness	34.61	13.53	−0.04	0.08	−0.02	−0.01	—	
6. Self-efficacy	34.35	5.94	−0.03	0.10 *	−0.10 *	−0.05	0.07	—

Note. *M* represents the mean; *SD* represents the standard deviation. * *p* < 0.05; ** *p* < 0.01.

**Table 3:** Mediation effect analysis results of victim self-disclosure valence and bystander intervention.

Effect	Effect Value	Boot SE	LLCI	ULCI	Ratio to Total Effect
Total Effect	−0.35	0.07	−0.21	−0.42	
Direct Effect	−0.16	0.07	−0.01	−0.19	45.71%
Indirect Effect	−0.19	0.03	−0.26	−0.13	54.29%

Note. LLCI: lower limit of 95% confidence interval; ULCI: upper limit of 95% confidence interval.

## Data Availability

The dataset in this research can be obtained upon request.
